# Idiopathic Acute Exudative Polymorphous Vitelliform Maculopathy: Insight into Imaging Features and Outcomes

**DOI:** 10.1155/2020/7254038

**Published:** 2020-01-28

**Authors:** Sónia Torres-Costa, Susana Penas, Ângela Carneiro, Renato Santos-Silva, Rodolfo Moura, Elisete Brandão, Fernando Falcão-Reis, Luís Figueira

**Affiliations:** ^1^Department of Ophthalmology, Centro Hospitalar Universitário de São João, Porto, Portugal; ^2^Department of Surgery and Physiology, Faculty of Medicine, University of Porto, Porto, Portugal; ^3^Department of Pharmacology and Therapeutics, Faculty of Medicine of the University of Porto, Porto, Portugal; ^4^Center for Drug Discovery and Innovative Medicines (MedInUP), University of Porto, Porto, Portugal

## Abstract

The authors describe imagiological findings in idiopathic exudative polymorphous vitelliform maculopathy. A 41-year-old woman complained of bilateral blurry vision. Best-corrected visual acuity was 20/20 bilaterally. Bilateral small serous neurosensory detachments in the fovea were seen at fundoscopy and confirmed by spectral-domain optical coherence tomography. Fluorescein angiography was unremarkable. Indocyanine green angiography presented discrete hyperfluorescent spots on the posterior pole. Later, more bleb-like lesions with a vitelliform appearance and hyperautofluorescent on blue fundus autofluorescence were detected. One year later, a complete resolution of the fluid was observed. To conclude, multimodal evaluation of patients with idiopathic exudative polymorphous vitelliform maculopathy is essential for the correct diagnosis of this disease.

## 1. Introduction

Idiopathic exudative polymorphous vitelliform maculopathy (IEPVM) is a rare disease, described by Gass in 1988 [1]. Only 20 additional idiopathic cases have been reported, but several paraneoplastic or infection-related cases have been described [1–4]. Age at presentation varies, ranging from 13 to 69 years, and both genders are affected [2]. This pathology is characterized by blurred vision, a mild decrease in visual acuity, and sometimes headaches [2]. Patients present with multiple yellow-white, morphologically variable lesions at the level of the retinal pigment epithelium (RPE) and serous neurosensory detachments (SND) with polymorphous subretinal yellowish deposits [1].

Although a decrease in photoreceptor function usually persists over time, this disorder has a good visual prognosis, with visual acuity improvement as resolution of macular edema progresses [2, 5].

Here, we describe the clinical findings and multimodal features of IEPVM during one year of follow-up.

## 2. Case Report

**Figure 1:**
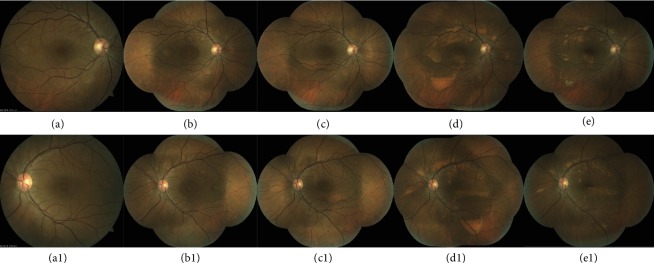
Right and left eye sequential color fundus photographs. At presentation, bilateral subfoveal serous retinal detachments were identified at the posterior pole (a and a1). One month after the onset of the visual symptoms, multifocal yellowish subretinal material was observed along the vascular temporal arcades (b and b1). Two months later, there is coalescence of the multiple lesions with progressive precipitation of vitelliform material (c and c1). Progressively, at the macular region, a large vitelliform detachment with a “pseudohypopyon” appearance and multiple vitelliform lesions with a honeycomb-like pattern appeared at the posterior pole. In this phase, the vitelliform material acquired a more yellowish coloration due to progressive accumulation (d and d1). One year after diagnosis, complete resolution of fluid with progressive reduction of curvilinear yellowish deposits could be seen.

**Figure 2:**
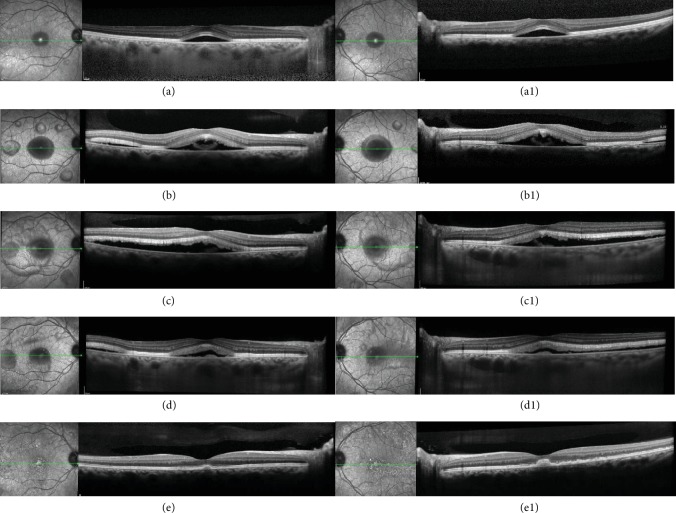
Sequential infrared and spectral domain optical coherence tomography (SD-OCT) of the right and left eyes. At presentation, bilateral subfoveal serous retinal detachment was present (a and a1). One month later, there was progression of bilateral macular subretinal fluid with the appearance of new small bleb-like serous retinal detachments along vascular arcades; SD-OCT showed a large serous neurosensory retinal detachment with remarkable thickening of the photoreceptor layer associated with the accumulation of amorphous material in the subretinal space (b and b1). Two months later, it is possible to observe the coalescence of the lesions. External limiting membrane and ellipsoid integrity was preserved (c and c1). At six months, SD-OCT revealed a marked reduction of the serous component in the detachments, and progressive shedding of the photoreceptor layer with more amorphous material accumulated in the subretinal space (d and d1). One year after diagnosis, a complete resolution of subretinal fluid with persistence of small vitelliform material deposits could be seen. It is possible to notice that the ellipsoid and external limiting membrane were slight disrupted.

**Figure 3:**
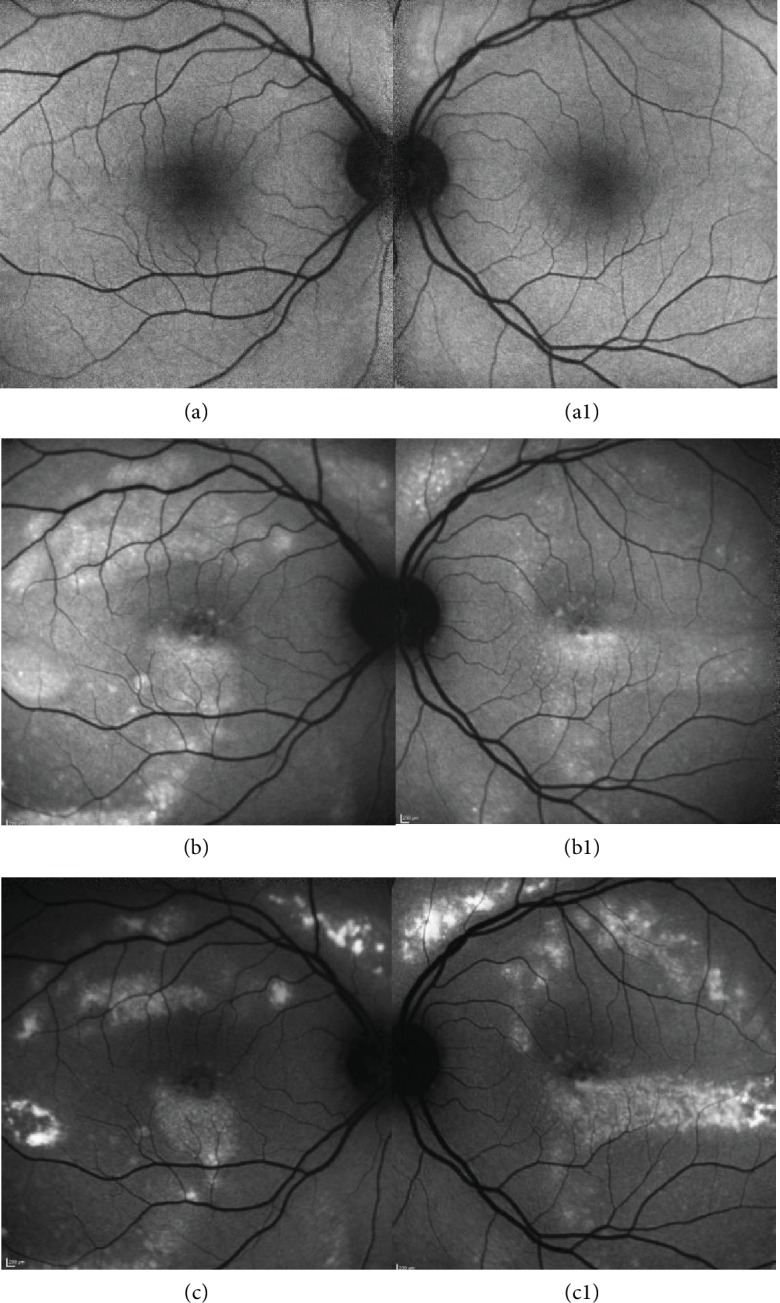
Right and left eye blue fundus autofluorescence (FAF) at presentation and eight months later. Initially, there was no significant evidence of hyperautofluorescent lesions in FAF (a and a1). As soon as the subretinal yellow-white vitelliform material starts to accumulate, FAF imaging shows the characteristic hyperautofluorescence of the polymorphous deposits. Frequently, vitelliform material precipitates along the inferior margins of the serous detachments, forming curvilinear deposits (b and b1). One year after diagnosis, FAF demonstrated a progressive reduction in hyperautofluorescence (c).

**Figure 4:**
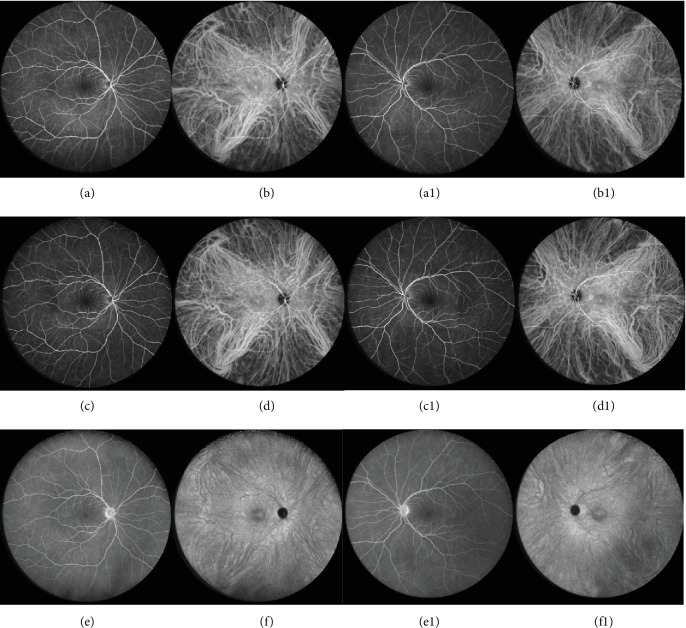
Right and left eye wide field fluorescein angiography (FA) and indocyanine green angiography (ICGA). At presentation, FA (a and a1) had an innocent aspect without any leakage or pooling evidence. ICGA (b and b1) presented very discrete hyperfluorescent spots in the posterior pole. Similarly, one month later, in early (c and c1) and late (e and e1) phases, FA remained relatively silent. Of note, ICGA revealed small hyperfluorescent points around vascular arcades in early (d and d1) and, more evidently, in late phases (f and f1).

We report the case of a 41-year-old white female patient who presented to our Emergency Department complaining of bilateral blurry vision for five days. Best-corrected visual acuity (BCVA) was 20/20 in both eyes. Anterior chamber examination and intraocular pressure were unremarkable. Fundoscopy showed small SND in the fovea bilaterally, with no signs of inflammation or optic nerve changes (Figures 1(a) and 1(a1)). Spectral-domain optical coherence tomography (SD-OCT) confirmed the foveal SND (Figures 2(a) and 2(a1)), with no internal reflectivity. Blue fundus autofluorescence (FAF) (Figures 3(a) and 3(a1)) and fluorescein angiography (Figures 4(a) and 4(a1)) were unremarkable and indocyanine green angiography presented very discrete hyperfluorescent spots in the posterior pole (Figures 4(b) and 4(b1)). An extensive workup that included blood analysis and cerebral and thoracic imaging excluded infectious, inflammatory, or neoplastic causes.

One month later, more bleb-like lesions were detected, acquiring a yellowish coloration, with a vitelliform appearance, that were hyperautofluorescent on FAF. SD-OCT showed multiple SND with internal hyperreflective deposits. Full-field electroretinography was normal, but electrooculography revealed an Arden ratio reduction to 1.38 in the right eye and 1.44 in the left eye. Six months later, the vitelliform detachments progressed to a “pseudohypopyon” appearance, and the fluid started to resolve. At one year of follow-up, complete resolution of the fluid with regression of deposits can be seen (Figures 1(e) and 1(e1)).

## 3. Discussion

Multimodal evaluation, a long follow-up time and high clinical suspicion are essential for the establishment of the correct diagnosis of patients with IEPVM. As we described in this clinical case, it was only possible to observe the appearance of vitelliform lesions, characteristically hyperautofluorescent on FAF, after one month of follow-up. Although the clinical features of IEPVM are being increasingly described in current literature, there is little information regarding its pathogenesis. More recently, it has been suggested that the autofluorescent material in IEPVM is derived not only from lipofuscin but also from indigestible components of phagocytized photoreceptor outer segments, which accumulate as a result of the lack of apposition of the retina to the RPE [1]. Some authors hypothesized that a possible variation in the chemical composition in the subretinal space is present in the course of the disease. According to this hypothesis, there is initially a transudate, resulting from the impaired RPE, that subsequently becomes enriched with lipofuscin and from photoreceptor outer segment shedding [1]. This theory is supported by FAF, in which a progressive increase in autofluorescence is noted as higher deposition of lipofuscin and indigestible components of phagocytized photoreceptor outer segments occurs.

Currently, there is no consensus as to whether prompt systemic or intraocular therapy with steroids or intraocular antivascular endothelial growth factor drugs could improve the clinical course. In fact, most results originated from isolated case reports and case series [1, 6].

Regardless of the chosen treatment, gradual visual recovery occurs over time, but electrophysiologic abnormalities may persist [1].

The presence of clinical and imagiological findings suggestive of exudative polymorphous vitelliform maculopathy warrants additional investigations to carefully exclude other underlying masquerading diseases and specifically paraneoplastic syndromes, as they are sometimes associated with this disorder [1].

Although IEPVM presents similarities with vitelliform macular dystrophy, no mutations in BEST1 or peripherin/RDS have been described [1]. Rarely, recurrence or secondary choroidal neovascularization can occur, reinforcing the importance of following these patients [1].
